# Drug Reaction With Eosinophilia and Systemic Symptoms Syndrome Preceding Toxic Epidermal Necrolysis Secondary to Sildenafil: A Case Report

**DOI:** 10.7759/cureus.63922

**Published:** 2024-07-05

**Authors:** Brooke Walterscheid, Maria Batchinsky, Michelle Tarbox

**Affiliations:** 1 Dermatology, Texas Tech University Health Sciences Center, Lubbock, USA

**Keywords:** south asian, sildenafil citrate, toxic epidermal necrolysis (ten), stevens-johnson syndrome (sjs), drug reaction with eosinophilia and systemic symptoms (dress) syndrome

## Abstract

Drug reactions with eosinophilia and systemic symptoms (DRESS) syndrome and Stevens-Johnson syndrome-toxic epidermal necrolysis (SJS-TEN) are reactive entities of aberrant cytotoxic immunologic reactions to exogenous medications. While they are conventionally seen as distinct, separate conditions, we present a case of a rare evolution of DRESS syndrome into SJS-TEN in the setting of simultaneous amoxicillin-clavulanate initiation and long-term sildenafil use in a 66-year-old South Asian female with a known history of prior DRESS syndrome and pulmonary arterial hypertension. We discuss the conditions leading to her unique clinical presentation and provide considerations for future clinical encounters.

## Introduction

Drug reaction with eosinophilia and systemic symptoms (DRESS) syndrome is a reactive eruption commonly caused by antiepileptics, antimicrobials, and other drugs such as allopurinol. It commonly presents with fever, facial swelling, diffuse and morbilliform rash, elevated eosinophil count, and evidence of organ system dysfunction [1]. Although triggered by a similar cohort of medications, Stevens-Johnson syndrome-toxic epidermal necrolysis (SJS-TEN) is a distinct spectrum of necrolytic skin and mucosal sloughing [2]. SJS involves a surface area below 10%, with TEN affecting over 30%. Although sources vary, DRESS syndrome and SJS have a mortality rate of around 10%, and the more severe TEN has a rate of up to 50%. Death from DRESS syndrome is typically because of acute fulminant hepatitis, while those with TEN succumb to sepsis and multi-organ system failure [1,2].

Although DRESS syndrome and SJS-TEN have independent etiologies, this case illustrates the atypical evolution of DRESS syndrome into SJS-TEN in the setting of simultaneous amoxicillin-clavulanate initiation and long-term sildenafil use. Sildenafil, a phosphodiesterase type 5 inhibitor, is a known treatment for erectile dysfunction and, less commonly, pulmonary arterial hypertension. Although amoxicillin-clavulanate is a typical inducer of both DRESS and SJS-TEN, sildenafil has only once been reported as inducing SJS-TEN when administered in high doses [3,4].

This report presents the second reported case of sildenafil-induced SJS-TEN and further discusses the distinct cutaneous and systemic findings of both DRESS and SJS-TEN observed in our patient. Ultimately, this case emphasizes cautious medication reintroduction and clinical monitoring in the post-drug reaction recovery period to immediately address any compounded sequelae, such as the unique progression of DRESS to SJS-TEN.

## Case presentation

A 66-year-old South Asian female with pulmonary arterial hypertension on sildenafil, type II diabetes, and chronic kidney disease (CKD) presented to our facility as a transfer from an outside hospital with a five-day progression of a diffuse, itchy rash beginning upon amoxicillin-clavulanate initiation, which initially prompted concern for SJS-TEN. She had been previously hospitalized for a complicated urinary tract infection and was placed on amoxicillin-clavulanate upon discharge. Notably, she had a history of prior DRESS syndrome secondary to beta-lactam use that was not made known to her care team at that time, likely because of her primary language being an Indian regional dialect.

**Figure 1:**
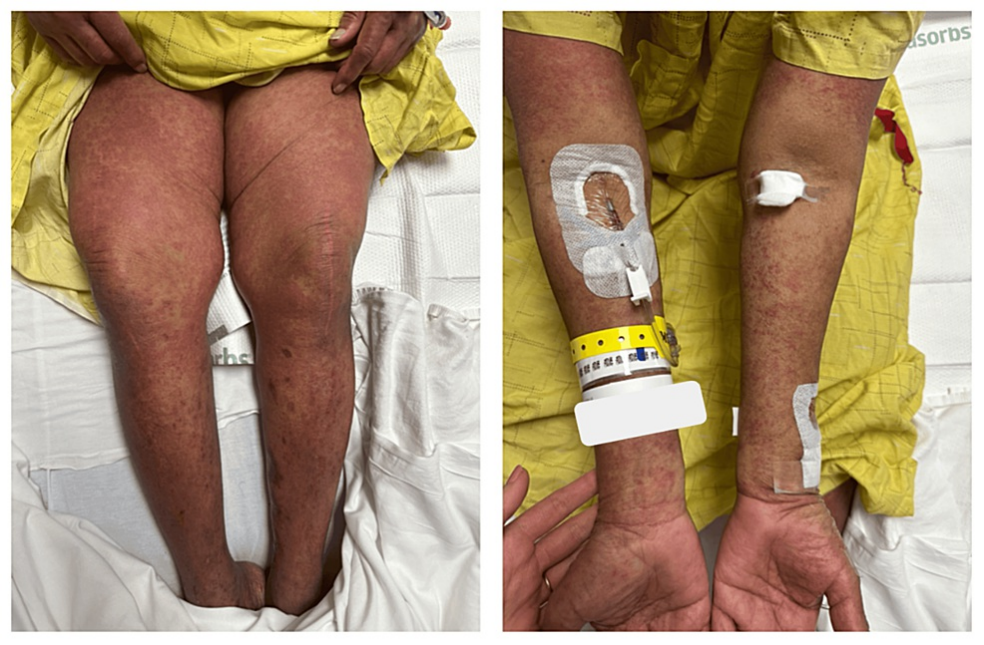
Morbilliform eruption of the bilateral extremities following the onset of facial edema on initial presentation, characteristic of drug reaction with eosinophilia and systemic symptoms (DRESS) syndrome.

Upon her arrival at our facility, dermatology was consulted and observed a typical morbilliform drug eruption on physical exam (Figure 1). Notably, she also had prominent facial edema with severe dryness of the cutaneous lip, but such findings did not extend to the mucosal lip. The oral cavity, tongue, eyes, and urethra were also uninvolved, thus quelling concerns for SJS-TEN. She additionally presented with other classic signs of DRESS syndrome, including eosinophilia of 3.5 K/uL (normal range: 0.0-1.0 K/uL), transaminitis with AST/ALT (alanine aminotransferase) 118/115 international units per liter (IU/L) (5-37/5-41 IU/L), and an acute kidney injury (AKI) on CKD with a blood urea nitrogen (BUN)/Cr of 1.6/37 mg/dL (6-20/0.5-1.2 mg/dL).

On admission, amoxicillin-clavulanate was immediately discontinued. The patient’s home medications, including carvedilol, pantoprazole, atorvastatin, hydroxyzine, and cetirizine, were all continued throughout her hospital stay. Sildenafil was prescribed for borderline low blood pressure. Given the extent of her rash and the associated lab findings, oral steroids were given in addition to topicals, and her rash steadily resolved. Her dry, cracked lips dramatically improved with topical emollients. Over the next five days, her rash markedly cleared, and the inpatient care team deemed her stable for discharge. She was scheduled with her primary care provider for labwork to monitor the down-trending AST/ALT and her nephrologist for the AKI on CKD. She was educated in her native dialect to strictly avoid beta-lactam antibiotics in the future, and this information was conveyed to her family as well.

**Figure 2:**
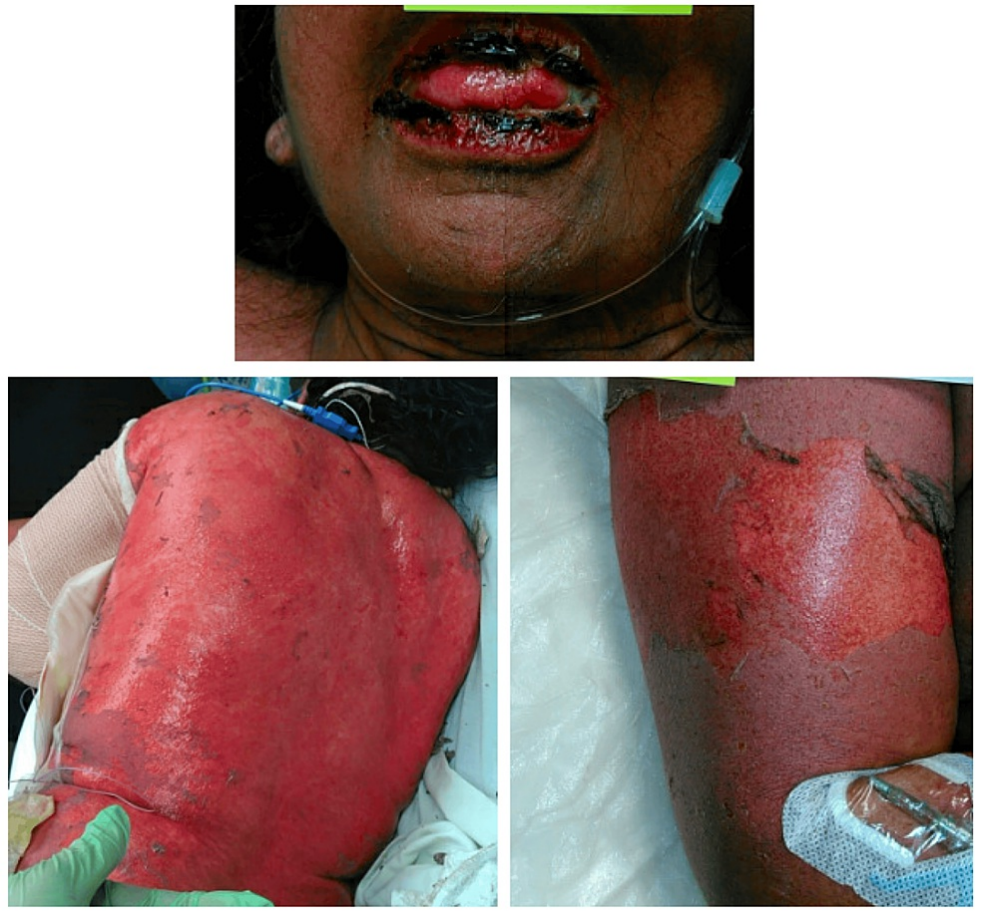
Mucosal and epidermal desquamation seen on readmission, clinically and histologically consistent with Stevens-Johnson syndrome-toxic epidermal necrolysis.

Within 24 hours after discharge, the patient returned to our facility in acute clinical decline. Dermatology was promptly consulted for distinct, new-onset cutaneous findings including the sloughing of the extremities with a positive Nikolsky’s sign and oral mucosal desquamation highly concerning for SJS-TEN (Figure 2). The patient’s son reported that the only medications she took the night before were sildenafil and prednisone. A careful examination of her medical record revealed that just before discharge on her most recent admission, the only medication reintroduced in that timeframe was high-dose sildenafil (20 mg TID), which was resumed after being held for the previous five days, during which time her clinical condition had improved. As declared in the adverse reaction section of the product’s monograph, sildenafil, especially in high doses, is an exceptionally rare cause of SJS-TEN [3].

Lesional punch biopsies were obtained immediately upon admission, during which repeat attempts were required as the epidermis would immediately detach from the dermis, further supporting our clinic diagnosis. H&E staining revealed acute full-thickness epidermal necrosis with dermal-epidermal separation and mild superficial perivascular lymphocytic infiltrate; non-lesional direct immunofluorescence for IgG, IgA, IgM, and C-3 was uniformly negative.

The burn specialty ICU was immediately notified, and the patient underwent sedation and intubation to protect her airway from progressive mucosal desquamation. Substantial pressor support was required shortly after that, which was complicated by efforts to maintain her fluid balance and renal function. The patient underwent RECELL application, an autologous cell harvesting method for skin grafting, after which a significant portion of the epidermis began to successfully re-epithelialize in the days following [5]. Despite this re-epithelialization, such high levels of pressor support were required over three hospital days that her distal extremities and small bowel developed overwhelming ischemic necrosis. Multisystem organ failure was grossly evident, and her family opted for compassionate extubation and comfort care measures.

## Discussion

Over two hospital admissions, this 66-year-old female’s case evolved from evident DRESS syndrome with a known inciting cause to, eight days later, a murkier presentation of SJS-TEN. While DRESS syndrome usually follows in the weeks after introducing the culprit drug, same-day symptoms may be seen upon reintroduction. It stands that our patient’s initial clinical presentation was most consistent with DRESS syndrome secondary to beta-lactam antibiotic use, especially given a known history of prior DRESS syndrome coupled with a classic gamut of symptoms that started the same-day amoxicillin-clavulanate was reintroduced and with the requisite laboratory findings. At that time, further scrutinization of her medication list was deferred once amoxicillin-clavulanate was deemed the probable culprit. As her condition improved upon cessation of the inciting medication, her hospital team deemed it safe to resume all her home medications.

As our patient resumed sildenafil on the day of her initial discharge, her AKI on CKD likely resulted in an accumulation of the medication’s metabolites, thus precipitating her rapid decline. The night of her discharge, she developed a burning oral sensation and took her prescribed prednisone and sildenafil with a glass of milk to cool the pain. A few hours later, her skin began to desquamate, and her family quickly took her back to their local emergency center, after which she was transferred back to our service. Only upon retrospective chart review during that second admission was it apparent that our patient likely clinically improved because of cessation of both amoxicillin-clavulanate and sildenafil.

At present, there is one reported case of sildenafil-induced SJS-TEN in the literature, in which a 67-year-old Middle Eastern man had taken one-time doses of sildenafil 100 mg and 200 mg for erectile dysfunction over two days and shortly thereafter developed oral pain and skin desquamation. It was suspected that polypharmacy affected the metabolic activity of his medications, resulting in this reaction. He remained hemodynamically stable and was successfully treated with supportive care and infliximab, a tumor necrosis factor-alpha (TNF-alpha) inhibitor [4]. Prior studies have demonstrated TNF-alpha as a primary mediator in the T-cell-driven cytotoxicity observed in SJS-TEN. Thus, TNF-alpha inhibitors have shown efficacy, as well as safety, in managing SJS-TEN. Intravenous immunoglobulin (IVIG) and cyclosporine have been employed as well, but their supporting data are not as sufficient as that seen with TNF-alpha inhibitors. However, these studies all conclude relatively small cohort sizes and would benefit from further validation by larger randomized controlled trials. Currently, there is neither a standardized protocol nor an FDA-approved treatment for SJS-TEN, leaving clinicians to determine treatment on a case-by-case basis [2].

As our patient's condition rapidly declined and she became hemodynamically unstable, the ICU deemed that she was not eligible for additional infusions; therefore, TNF-alpha inhibitor therapy was considered, but ultimately deferred. While over 1.3 million patients were taking sildenafil in 2021 for either pulmonary arterial hypertension or erectile dysfunction, there are now only two reported cases of SJS-TEN over decades, rendering it a generally safe medication [6]. Albeit rare, it is still important to educate patients on the potential for a drug-related eruption, such as DRESS syndrome or SJS-TEN, no matter the prescription. Further, it is critical that patients immediately stop all nonessential medications at the first onset of a drug-related rash, as early cessation is paramount in decreasing mortality.

## Conclusions

Our patient’s distinct beta-lactam-induced DRESS syndrome preceding SJS-TEN from sildenafil is a highly unusual presentation, wherein swift dermatologic evaluation is necessary. As many drug eruptions present with overlapping features, clinicians should employ careful physical examination, detailed history taking, and close lab monitoring in narrowing a differential diagnosis. This case emphasizes the importance of educating patients on potential, though rare, side effects and in stopping even seemingly benign nonessential medications immediately upon the onset of a potential drug eruption. Such education must meet the patient’s level of understanding, especially when faced with language barriers. When reintroducing medications, patients should be closely monitored for new-onset symptoms. As sildenafil is widely prescribed, clinicians should be aware of this exceptionally rare, but devastating side effect, as this case is only the second reported of sildenafil-induced SJS-TEN. If the clinical presentation is consistent with SJS-TEN, patients should immediately be assessed by both burn intensive care and dermatology. TNF-alpha inhibitors may be considered in those patients amenable to infusion therapy, although larger studies are needed to validate its efficacy against other treatments, such as IVIG or cyclosporine.
